# Nitric Oxide-S-Nitrosylation and Its Role in Neuroinflammation Associated with Neuropsychiatric Conditions

**DOI:** 10.3390/ijms27083615

**Published:** 2026-04-18

**Authors:** Fabiola Sánchez, Tania Koning

**Affiliations:** 1 Instituto de Inmunología, Facultad de Medicina, Universidad Austral de Chile, Valdivia 5110566, Chile; 2 Centro Interdisciplinario de Estudios del Sistema Nervioso (CISNe), Universidad Austral de Chile, Valdivia 5110566, Chile; 3 Instituto de Alta Investigación, Universidad de Tarapacá, Arica 1000000, Chile; tkoning@academicos.uta.cl

**Keywords:** nitric oxide, S-nitrosylation, blood–brain barrier, neuropsychiatric conditions, endothelial permeability, inflammation, stress

## Abstract

Neuropsychiatric conditions constitute a major and growing global health burden, with prevalence rates that continue to rise worldwide. Although these disorders have traditionally been studied primarily from a neuronal perspective, accumulating evidence indicates that immune dysregulation and inflammatory processes play a central role in their pathophysiology. In this review, we advance the hypothesis that nitric oxide (NO)-mediated alterations in blood–brain barrier (BBB) integrity represent a critical mechanistic link between inflammation and central nervous system dysfunction in neuropsychiatric disorders. NO is a gaseous multifunctional signaling molecule involved in vascular homeostasis and immune responses, and its dysregulated production, together with aberrant protein S-nitrosylation, has been implicated in several neuropsychiatric conditions. However, the specific mechanisms by which NO signaling contributes to BBB dysfunction remain incompletely defined. Here, we synthesize current evidence supporting a role for NO-dependent vascular and inflammatory pathways in BBB disruption and discuss how these processes may contribute to the onset and progression of neuropsychiatric conditions. Clarifying these mechanisms may provide novel insights into disease pathogenesis and identify therapeutic targets aimed at preserving BBB integrity and limiting neuroinflammation.

## 1. Introduction

Neuropsychiatric conditions are recognized as leading causes of burden disease with no sign of reduction in the burden since 1990 [1]. In 2019, 1 in every 8 people around the world were living with a mental disorder [1]. According to the World Health Organization (WHO), since March 2020, mental health problems have increased worldwide because of the coronavirus-19 (COVID-19) pandemic. In the United States it is estimated that more than one in five adults live with a mental disorder [2]. It not only constitutes a problem for people that suffer from the disease but also for their entire social circle. Neuropsychiatric conditions are characterized by a significant disturbance in an individual’s cognition, emotional regulation and behavior which prevents normal social functioning and makes people with the disease to be highly stigmatized. In fact, the Lancet Commission on global mental health and sustainable development established mental health as a human right, essential to the development of all countries [1].

While traditional studies have primarily focused on neuronal deficiencies, researchers have begun to recognize the importance of the immune system and inflammatory processes in neuropsychiatric conditions. In recent years, there has been growing evidence suggesting that inflammation and the breakdown of the blood–brain barrier (BBB) play significant roles in the development and progression of various neuropsychiatric conditions [3,4,5,6,7,8].

The BBB protects the brain from external insults forming a semi-permeable barrier between the systemic circulation and the brain [9]. This structure maintains a stable brain environment and is essential for effective synaptic communication and the preservation of neuronal health and structure [10]. Recently, the BBB has gained importance as a dynamic interface critical to brain function and has become a focal point in research related to the development of various central nervous system (CNS) disorders [3,4,5,6,7,8].

Nitric oxide, (NO), is a key molecule regulator of many physiological functions linked to critical roles in processes like inflammation, where it modulates vascular functions either via traditional signaling pathways or through protein modifications such as S-nitrosylation. Excessive NO production and aberrant S-nitrosylation of proteins have been implicated in the pathogenesis of several neuropsychiatric conditions [11,12,13]. NO is responsible for increased vascular permeability in various tissues; however, its role in BBB vascular permeability has not been clarified. In this review we will analyze the evidence that supports that NO leads to disruption of the BBB contributing to neuropsychiatric conditions.

## 2. Nitric Oxide and S-Nitrosylation

NO is a signaling molecule that regulates a wide range of physiological processes [14,15,16]. It is synthesized by three nitric oxide synthase (NOS) isoforms: endothelial (eNOS), inducible (iNOS), and neuronal (nNOS). While eNOS and nNOS are constitutively expressed, iNOS is induced under pro-inflammatory conditions [16]. NO signals through activation of the soluble guanylyl cyclase–protein kinase G pathway and through S-nitrosylation, a reversible post-translational modification involving the covalent attachment of NO to cysteine residues [17,18,19].

S-nitrosylation mediates a substantial proportion of NO-dependent signaling by regulating protein localization, interactions, and activity [17,18,19,20,21,22,23]. Although NO is diffusible, its short half-life makes the spatial and temporal context of its production critical, such that S-nitrosylation patterns depend on the specific NOS isoform expressed within a given cell type [24,25,26,27]. In addition, S-nitrosylation can be propagated through transnitrosylation reactions and is tightly controlled by denitrosylating systems, including S-nitrosoglutathione reductase (GSNO) and thioredoxin [19,28,29].

In the central nervous system, NO participates in processes such as synaptic plasticity, neurotransmission, learning, and memory [30,31,32]. Dysregulated NO production and aberrant S-nitrosylation have been implicated in multiple neuropsychiatric and neurodegenerative disorders [13,33,34,35,36,37,38,39]. However, whether NO and S-nitrosylation directly contributes to BBB dysfunction remains unclear, and the molecular mechanisms linking NO signaling to BBB breakdown are still incompletely understood.

## 3. Neurovascular Unit and BBB

The concept of neurovascular unit (NVU) emerged from the Stroke Progress Review Group meeting convened by the National Institute of Neurological Disorders and Stroke at the National Institutes of Health (July 2001). This framework was proposed to highlight the close developmental, structural, and functional interdependence between neural cells and the cerebral microvasculature, as well as their coordinated responses to physiological challenges and injury [40].

**Figure 1:**
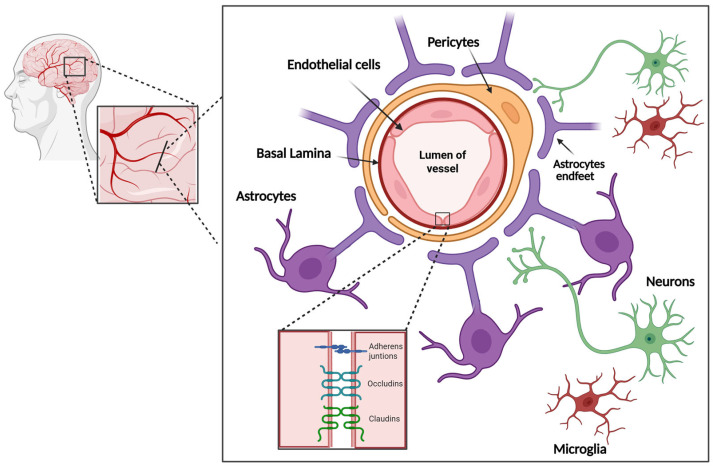
The neurovascular unit (NVU). The endothelial cells, whose intracellular spaces are sealed by tight and adherens junctions, are surrounded by mural cells, such as pericytes and astrocytes. Both endothelial and mural cells are enveloped by basal laminae, providing structural and functional support to the blood–brain barrier (BBB). Astrocytic endfeet cover nearly the entire surface of blood vessels and directly interact with both endothelial cells and pericytes. Neurons engage with endothelial cells and astrocytes, and their activity can modulate vascular tone either directly on endothelial cells or indirectly via astrocytes. Microglial processes establish dynamic and transient connections with the vasculature, astrocytes, and neurons, and contribute to the NVU (modified from [43]).

The NVU is composed of neurons, astrocytes, microglia, endothelial cells, pericytes and extracellular matrix components, as shown in Figure 1. These cells, through their intimate anatomical and chemical relationship, detect the needs of neuronal supply and trigger necessary responses (vasodilation or vasoconstriction) for such demands [41,42]. In this context, the NVU should be understood as a broader regulatory and signaling unit, whereas the BBB refers more specifically to the endothelial barrier itself, defined by endothelial cells and their intercellular junctions that restrict paracellular and transcellular permeability. Importantly, BBB properties are not intrinsic to endothelial cells alone but are dynamically controlled by the surrounding NVU. NO participates in this crosstalk by acting within the NVU to modulate vascular tone, endothelial junction organization, and inflammatory signaling, thereby indirectly shaping BBB integrity.

### 3.1. Endothelial Cells

The BBB is a dynamic and highly regulated interface maintained by the coordinated activity of endothelial cells, pericytes, astrocytes, and microglia. Endothelial cells lining brain capillaries constitute the structural core of the BBB and display a highly specialized phenotype characterized by a restrictive glycocalyx, minimal vesicular trafficking, and well-developed tight and adherens junctions that ensure selective molecular exchange between the blood and brain parenchyma [44,45,46,47]. Tight junctions are formed by transmembrane proteins such as claudins, occludin, and junctional adhesion molecules, which are linked to the actin cytoskeleton via scaffolding proteins including ZO-1, ZO-2, and ZO-3, while adherens junctions are primarily mediated by VE-cadherin and associated catenins, providing endothelial cohesion and junctional regulation [45,46,47,48,49]. BBB integrity further depends on continuous signaling from astrocytes, pericytes, and neurons, as well as integrin-mediated interactions with the basement membrane, which provides mechanical support and transduces signals essential for junctional stability [50,51]. The specific role of NO in BBB will be revised in detail in posterior sections.

### 3.2. Pericytes

Pericytes closely associate with endothelial cells along brain capillaries and are critical for BBB maintenance and vascular stability through direct cell–cell contacts and paracrine signaling [52]. They regulate angiogenesis, capillary diameter, cerebral blood flow, and clearance of toxic metabolites [52,53,54,55]. In the adult brain, pericyte dysfunction—rather than overt detachment—is increasingly recognized as a contributor to BBB impairment in neurological disorders such as Alzheimer’s disease, dementia, amyotrophic lateral sclerosis, and stroke [52,56,57,58,59,60,61,62]. Increased attention has been given to the role of pericytes in neuroinflammatory signaling within the neurovascular unit. Although still less well characterized, pericytes can produce NO, undergo protein S-nitrosylation, and secrete cytokines and chemokines in response to inflammatory stimuli such as lipopolysaccharide [63].

### 3.3. Astrocytes

Astrocytes play a central role in BBB establishment and maintenance through their perivascular endfeet, which nearly completely ensheath cerebral capillaries [45,46,64,65]. Astrocyte-derived signals induce and stabilize endothelial tight junctions, and astrocytic loss or dysfunction is associated with increased BBB permeability [17,57,58,59,60,61]. Regulation of BBB properties involves both soluble mediators, including extracellular vesicles, and direct cell–cell interactions with endothelial cells [42,43,66,67,68,69,70,71]. Under inflammatory or stress conditions, astrocytes can adopt a reactive phenotype and release factors such as matrix metalloproteinases and VEGF-A, leading to disruption of junctional proteins and extracellular matrix components, BBB breakdown, and vasogenic edema [67,72,73,74]. Reduced astrocytic vascular coverage has been reported in major depressive disorder and schizophrenia, highlighting the relevance of astrocyte-mediated BBB dysregulation in neuropsychiatric disease [75,76,77,78,79,80].

### 3.4. Microglia

Microglia are the resident immune cells of the central nervous system and are essential for immune surveillance and tissue homeostasis [43]. Upon injury or stress, they become activated and display marked phenotypic plasticity. While acute activation can be protective, chronic activation promotes a pro-inflammatory environment characterized by the sustained release of cytokines, reactive oxygen species, and NO, which can compromise BBB integrity [81,82]. Altered microglial activation has been reported in psychiatric disorders such as schizophrenia and major depressive disorder, implicating dysregulated microglial NO signaling in disease-associated BBB alterations [83,84,85,86].

## 4. NO and Its Role in the Breakdown of the BBB

Evidence supports the role of NO in the breakdown of the BBB. NO donors increase BBB permeability to different extents, depending on the specific NO donor used [87,88,89], while NOS inhibition blocks the permeability response induced by histamine and infection with *E. coli* [90,91]. In agreement with these findings, eNOS knock-out (KO) mice fail to develop BBB leakage in response to septic plasma and VEGF [91,92]. At the molecular level, NO-mediated BBB disruption has been associated with the dissociation of occlaudin from tight junction complexes, reduced expression of occludin, ZO-1 and ZO-2, and upregulation of matrix metalloproteinase-9 (MMP-9), collectively favoring junctional destabilization and extracellular matrix degradation [92,93].

Nevertheless, some studies report apparently opposing effects, showing that NOS inhibition worsens BBB damage in traumatic brain injury models [24], or that eNOS KO mice exhibit enhanced BBB disruption and elevated intracranial pressure in experimental meningitis [94]. These discrepancies likely reflect differences in disease context, inflammatory burden, timing, and NOS isoform involvement. Importantly, the biological actions of NO are highly concentration-dependent and closely linked to its cellular source and subcellular localization [42,54,55,95]. Under basal conditions, low levels of eNOS-derived NO are generally considered protective, supporting cerebral blood flow, endothelial survival, and junctional stability. In contrast, acute and sustained inflammatory stimulation induces a rise in NO production via eNOS and iNOS activation, respectively. Sustained iNOS activation promotes peroxynitrite formation and pathological nitrosative stress, which may result in persistent BBB breakdown. Similar NO-dependent, context-specific effects have been described in leukocyte adhesion in peripheral tissues [15,25].

Thus, the apparent controversy in literature largely reflects a shift from basal NO signaling to an elevation in NO concentration from eNOS and iNOS activation, with outcomes further modulated by disease stage, inflammatory burden, and intervention timing.

In other vascular territories different from brain, we have demonstrated the key role of eNOS in hyperpermeability using eNOS KO mice and eNOS depleted endothelial cells [26,27]. In both models the increase in permeability in response to pro-inflammatory agonists is inhibited. Mechanistically, pro-inflammatory stimuli (including VEGF, IL-8, TNF-α, PAF, and tumor-derived factors) induce eNOS-mediated S-nitrosylation of adherens junction proteins such as VE-cadherin, p120-catenin, and β-catenin, leading to their phosphorylation, junctional disassembly, and internalization, ultimately increasing endothelial permeability [20,21,96,97,98]. We also demonstrated that S-nitrosylation of vasodilator stimulated phosphoprotein (VASP) a focal adhesion-associated protein, contributes to permeability increases, likely by regulating endothelial interactions with the basal lamina through focal adhesion reorganization [99].

Importantly, eNOS subcellular localization is a critical determinant of its permeability-inducing effects in peripheral vascular territories. Endothelial cells expressing eNOS mutants targeted to different compartments generate similar amounts of NO upon stimulation, however only cytosolic eNOS localization promotes S-nitrosylation-dependent increases in permeability [14,20,23,27,99,100,101]. These findings are consistent with earlier reports showing that eNOS preferentially S-nitrosylates proteins within its immediate subcellular environment [102].

Whether similar S-nitrosylation-dependent mechanisms operate at the BBB remains unknown. BBB integrity relies predominantly on tight junctions, such as claudins and occludin, rather than solely on adherens junctions. Although adherens junction proteins like VE-cadherin contribute to endothelial stability and can influence tight junction organization, there is currently no direct evidence demonstrating eNOS-mediated S-nitrosylation of tight junction proteins at the BBB. Consequently, most mechanistic data linking S-nitrosylation to increased permeability derive from peripheral vasculature and cannot be directly extrapolated to the BBB. This complexity is further increased by the contribution of other neurovascular unit cell types. Whether NO or S-nitrosylation modulates pericyte, astrocyte, or microglial functions in ways that favor BBB disruption remains largely unexplored.

## 5. Translational Comparison of Experimental and Clinical Approaches to Assess BBB Permeability

When interpreting BBB permeability data, it is important to consider the methodological differences between animal models and clinical detection techniques, as these differences strongly influence the type of information obtained. In experimental studies, BBB permeability is frequently evaluated using tracer extravasation assays, such as Evans blue or other low-molecular-weight dyes. These approaches are highly sensitive and provide a direct measure of barrier leakage in brain tissue, mainly reflecting albumin-associated macromolecular extravasation and marked disruption of tight junctions. However, they are invasive, usually terminal, and capture permeability as a cumulative endpoint rather than a dynamic process [103].

In clinical settings, BBB permeability is mainly assessed using non-invasive imaging techniques, particularly dynamic contrast-enhanced magnetic resonance imaging (DCE-MRI). This method estimates BBB permeability indirectly by analyzing the kinetics of gadolinium-based contrast agents and generates parameters such as K_trans_ which depend not only on endothelial permeability but also on cerebral blood flow and modeling assumptions. Although DCE-MRI allows regional and longitudinal assessment of BBB alterations, its sensitivity is lower compared with tracer-based assays used in animal models, especially when BBB changes are mild or transient [103,104].

Therefore, discrepancies between preclinical and clinical findings often reflect methodological rather than biological differences. Tracer extravasation assays mainly detect leakage of protein-bound macromolecules, whereas DCE-MRI is more sensitive to changes in permeability to small solutes. Recognizing these distinctions is essential for improving translational interpretation of BBB studies and for cautious comparison across species and techniques [103,104].

## 6. Stress as a Major Risk Factor for Neuroinflammation and Neuropsychiatric Conditions

Stress is a well-established environmental risk factor for neuropsychiatric disorders, including depression, anxiety, and schizophrenia [105,106,107]. Experimental and clinical studies suggest that stress may contribute to disease pathogenesis, at least in part, through BBB disruption [108]. Chronic stress promotes inflammatory processes that impair BBB integrity and can precipitate behavioral changes associated with depressive-like phenotypes [109].

Stress-induced inflammation arises through multiple, interconnected pathways. First, stress exposure leads to the release of damage-associated molecular patterns (DAMPs), such as high-mobility group box 1 (HMGB1), heat shock proteins, and S100 proteins, which activate Toll-like receptors on microglia and astrocytes, triggering cytokine production [109,110]. These cytokines further amplify glial activation and sustain neuroinflammation [111,112]. Second, stress activates the hypothalamic–pituitary–adrenal (HPA) axis, resulting in glucocorticoid release [113,114]. While glucocorticoids are typically anti-inflammatory, chronic stress induces HPA axis dysregulation and glucocorticoid resistance, leading to impaired immune regulation and a shift toward pro-inflammatory states [115]. Third, activation of the sympathetic–adrenal–medullary (SAM) axis promotes norepinephrine release, enhancing immune cell activity and cytokine production in peripheral tissues [112,113,116]. These circulating cytokines can access the brain and contribute to central inflammatory responses.

Under chronic stress conditions, elevated levels of pro-inflammatory cytokines (including IL-1β, IL-6, TNF-α, and IFN-γ) in both peripheral blood and brain tissue disrupt endothelial tight and adherens junctions, promote oxidative stress, and impair astrocyte and pericyte interactions with the BBB. BBB disruption, in turn, facilitates immune cell infiltration and further cytokine entry into the CNS, creating a feed-forward loop that amplifies neuroinflammation [117,118]. Importantly, these pro-inflammatory cytokines activate intracellular signaling cascades in multiple cell types of the NVU including endothelial cells, astrocytes, microglia, and pericytes that stimulate the expression and activity of NOS, thereby increasing NO production [119]. In addition to contributing to oxidative and nitrosative stress, these inflammatory mediators alter neuronal function, impair synaptic plasticity, disrupt neurotransmission, and inhibit neurogenesis, ultimately contributing to depressive-like symptoms [42,109].

In the CNS, while NO has been implicated in BBB disruption, whether S-nitrosylation of specific targets within the neurovascular unit contributes to BBB permeability remains unresolved. Increased NO production induced by inflammatory signaling may promote S-nitrosylation of proteins involved in junctional stability, cytoskeletal organization, and cell–cell communication within the NVU. However, direct evidence identifying S-nitrosylated targets that regulate BBB permeability is still limited. Therefore, S-nitrosylation should be viewed as a biologically plausible but not yet fully validated mechanism linking inflammation, stress, and BBB dysfunction. This framework highlights current knowledge gaps and underscores the need for BBB-focused proteomic and in vivo studies to identify relevant S-nitrosylated targets and clarify their contribution to neuroinflammation and psychiatric-relevant pathophysiology.

## 7. Major Depressive Disorder (MDD)

The pathophysiology of MDD is multifactorial and incompletely understood. Dysregulation of monoaminergic neurotransmission, impaired neuroplasticity, HPA axis dysfunction, neuroendocrine alterations, oxidative stress, and immune activation have all been implicated [120]. However, despite decades of research, causal relationships between these processes remain difficult to establish. This uncertainty is reflected in the limited therapeutic innovation in antidepressant treatment since the 1950s, which has largely focused on enhancing serotonergic and noradrenergic signaling, with a substantial proportion of patients showing inadequate clinical response [120]. These limitations have led to interest in alternative and complementary pathophysiological frameworks, including inflammation and neurovascular dysfunction.

### 7.1. BBB Alterations in MDD

Emerging evidence from postmortem, neuroimaging, and preclinical studies suggests that BBB alterations may occur in MDD, although their causal role remains unclear. Postmortem analyses have reported reduced expression of tight junction proteins, particularly claudin-5, in mood-related brain regions such as the nucleus accumbens and hippocampus [121,122], while increased serum claudin-5 levels in patients have been proposed as indirect markers of BBB disruption [123]. Neuroimaging studies have also indicated increased BBB permeability in regions including the thalamus, caudate, and hippocampus, correlating with depression severity, although methodological limitations restrict definitive conclusions [124]. Similarly, animal models of chronic stress show increased BBB permeability and reduced junctional protein expression in limbic and cortical regions [108,125,126], supporting the idea that stress can impair neurovascular integrity, though whether BBB dysfunction is a cause or consequence of depressive pathology remains unresolved [127].

### 7.2. Endothelial NO Signaling and Vascular Permeability

Endothelial cells are a major source of NO within the neurovascular unit and play a central role in regulating vascular tone, cerebral blood flow, and BBB permeability. Under physiological conditions, eNOS-derived NO contributes to vascular homeostasis and neurovascular coupling. However, under stress or inflammatory conditions, dysregulated NO production may contribute to endothelial dysfunction and alterations in junctional stability.

In this context, vascular endothelial growth factor (VEGF) signaling has emerged as a potential mediator linking stress exposure to BBB permeability. In chronic restraint stress models, increased BBB permeability and anhedonic behavior were prevented by pharmacological inhibition of VEGFR-2 [127]. Because VEGF signaling is closely coupled to eNOS activation and NO production, these findings raise the possibility that NO-dependent endothelial signaling contributes to stress-induced neurovascular alterations. Nevertheless, the relevance of these mechanisms to human MDD remains uncertain and may be limited to specific disease subtypes.

### 7.3. Astrocytic NO Signaling and BBB Regulation

Astrocytes represent an important source of NO signaling within the neurovascular unit. These cells express all three NOS isoforms (eNOS, nNOS, and iNOS) and contribute to NO-mediated communication between neural and vascular compartments [128,129]. Astrocytic endfeet closely interact with the endothelial basal lamina and play a key role in BBB maintenance, largely through the polarized distribution of proteins such as aquaporin-4 (AQP-4) and Kir4.1 [46,64,130,131]. Notably, astrocytic alterations have been consistently described in both patients with MDD and animal models of depressive-like behavior, including reduced astrocyte density and diminished endfeet coverage of cerebral vessels [123,132,133].

Reduced expression of AQP-4, a key determinant of astrocyte polarity and water homeostasis, has been associated with increased vascular permeability and depressive-like phenotypes [128,129,134,135,136]. Importantly, NO has been shown to modulate AQP-4 expression and astrocyte morphology under inflammatory conditions [137,138,139], suggesting that NO-dependent signaling may influence astrocyte–vascular interactions and BBB stability. Functional evidence supports the importance of AQP-4 for neurovascular integrity: AQP-4 knockout mice display BBB hyperpermeability, altered astrocytic endfoot morphology, and more pronounced depressive-like behaviors [134,135,136]. In line with these findings, reduced AQP-4 expression at astrocytic endfeet has been reported in postmortem brain tissue from individuals with MDD and in animal models of depressive-like behavior, correlating with loss of tight junction components and increased vascular permeability [76,80,128]. One potential mechanism linking NO signaling to astrocytic regulation of BBB function is the S-nitrosylation that can regulate protein trafficking, localization, and activity. Although S-nitrosylation of AQP-4 has not yet been reported, other aquaporins, including AQP-1 and AQP-5, have been identified as S-nitrosylated proteins in cardiac and neural tissues [140,141]. These observations raise the possibility that NO-dependent post-translational regulation may also occur for AQP-4.

Similarly, connexin-43, a protein involved in astrocyte morphology and endfoot organization, can undergo S-nitrosylation in astrocytes and has been implicated in depression [142,143,144], although whether this modification affects BBB permeability or astrocyte–endothelial interactions remains unknown.

### 7.4. Microglial NO Signaling and Neurovascular Inflammation

Microglia-derived NO represents another potential contributor to neurovascular alterations in MDD. Activated microglia produces substantial amounts of NO during inflammatory responses, which may influence endothelial function and BBB integrity. Interestingly, antidepressants such as selective serotonin reuptake inhibitors (SSRIs) and serotonin–norepinephrine reuptake inhibitors (SNRIs) have been shown to reduce microglial NO production and inflammatory signaling in vitro [145,146,147,148,149]. These findings suggest that modulation of NO-related pathways may contribute, at least in part, to the therapeutic effects of these drugs. However, the in vivo relevance of microglial NO signaling for BBB function in MDD remains to be established.

### 7.5. Integrative Perspective

Taken together, NO signaling across multiple cell types of the neurovascular unit—including endothelial cells, astrocytes, and microglia—emerges as a context-dependent modulator of neurovascular and inflammatory processes in MDD. Although alterations in NO production and nitric oxide synthase activity are well documented, direct mechanistic links between NO-driven S-nitrosylation and BBB dysfunction in depression have not yet been demonstrated. Consequently, S-nitrosylation should currently be considered a biologically plausible but still unproven mechanism contributing to neurovascular pathology in MDD, highlighting the need for targeted proteomic and in vivo studies to identify relevant S-nitrosylated proteins within the BBB.

## 8. Schizophrenia

Schizophrenia is a severe and disabling psychiatric disorder affecting up to 1% of the global population and is among the leading causes of disability worldwide [150]. The disease typically manifests in late adolescence or early adulthood, a critical developmental period characterized by synaptic pruning and circuit refinement [151]. Clinically, schizophrenia is defined by a heterogeneous constellation of symptoms that can be broadly classified into positive symptoms, negative symptoms, and cognitive deficits reflecting widespread dysfunction across neural systems [152].

Despite extensive investigation, schizophrenia is best conceptualized as a complex neurodevelopmental disorder arising from the interaction of genetic vulnerability and environmental factors [153,154]. Molecular and transcriptomic studies increasingly imply immune and oxidative stress pathways in disease-relevant brain regions. However, how these molecular alterations translate into neurovascular dysfunction or BBB impairment remains poorly defined.

### 8.1. BBB Disruption in Schizophrenia

Multiple lines of evidence suggest BBB alterations in schizophrenia, although their extent and functional significance remain debated [155]. Elevated CSF/serum albumin ratios (QAlb) provide indirect evidence of barrier dysfunction [156], while postmortem studies reveal changes in capillary density and endothelial markers. However, these findings do not consistently demonstrate BBB breakdown.

Advanced neuroimaging techniques, such as DCE-MRI and ASL, have revealed regional alterations in BBB permeability and cerebral perfusion [157]. Interpretation of these findings is challenging, as they may reflect subtle, region-specific changes rather than global barrier failure.

### 8.2. Endothelial NO Signaling and Vascular Regulation

Genetic studies implicating claudin-5 polymorphisms [158,159,160,161] and experimental models showing size-selective BBB leakage in claudin-5-deficient mice [162] suggest that BBB alterations in schizophrenia may be subtle and functionally selective. These data support the concept of partial or regulated BBB dysfunction rather than complete barrier collapse. Proteolytic mechanisms, including increased MMP-9 activity, may further contribute to barrier instability [163,164,165,166]. However, whether MMP-9-mediated remodeling directly drives BBB dysfunction in schizophrenia or reflects secondary inflammatory processes remains unresolved. Also, increased BBB permeability associated with reduced expression of ZO-1 has been observed in human-induced pluripotent stem cells derived from schizophrenia patients [7]. Altered NO signaling has been repeatedly reported in schizophrenia, although findings are inconsistent, with both increased and decreased NO levels described [167,168]. VEGF–VEGFR2 signaling, which activates eNOS, has been proposed as a potential contributor to endothelial dysfunction [169,170,171]. Based on evidence from peripheral vascular systems, this pathway could theoretically promote S-nitrosylation of junctional proteins and increase permeability [20,21,96,97,98]. However, direct evidence that this mechanism operates at the BBB in schizophrenia is currently lacking.

### 8.3. Astrocytic NO Signaling and BBB Regulation

Astrocytes play a key role in maintaining BBB integrity through their perivascular endfeet and interactions with endothelial cells. Astrocytic abnormalities have been described in schizophrenia, including structural and functional alterations that may disrupt astrocyte–vascular communication [79]. Because astrocytes express multiple nitric oxide synthase isoforms, altered astrocytic NO signaling could potentially influence endothelial junctional stability and neurovascular coupling. However, the contribution of astrocyte-derived NO or S-nitrosylation of astrocytic proteins to BBB dysfunction in schizophrenia remains largely unexplored.

### 8.4. Microglial NO Signaling and Neurovascular Inflammation

Excessive microglial activation is also associated with the pathophysiology of schizophrenia. Postmortem studies of the brains of schizophrenia patients consistently demonstrate elevated microglial activation disrupting BBB through various mechanisms [157]. These include the activation of iNOS, the promotion of ROS synthesis, inducting cyclooxygenase 2 (COX-2) expression within the neurovascular unit, and the upregulation of pro-inflammatory cytokines and matrix metalloproteinases [172]. Increased BBB permeability may, in turn, allow peripheral immune factors to infiltrate CNS. This results in the activation of glial cells, further cytokine production, and a cycle of neuroinflammation [173]. Nevertheless, whether microglia-derived NO directly regulates BBB permeability in schizophrenia remains unclear.

### 8.5. Integrative Perspective

Overall, current evidence suggests that BBB dysfunction in schizophrenia is subtle, regionally heterogeneous, and mechanistically complex. Although alterations in endothelial signaling, inflammatory pathways, and NO metabolism have been reported, direct mechanistic links between nitric oxide-dependent S-nitrosylation and BBB permeability remain largely speculative. Future studies integrating neurovascular imaging, molecular analyses, and redox proteomics will be necessary to clarify whether S-nitrosylation contributes to BBB regulation in schizophrenia.

## 9. Autism Spectrum Disorder

Autism Spectrum Disorder (ASD) is a neurodevelopmental condition characterized by persistent impairments in social communication and social interaction, together with restricted and repetitive patterns of behavior, interests, or activities. According to the Diagnostic and Statistical Manual of Mental Disorders, Fifth Edition, symptoms typically emerge in early childhood and are associated with long-term developmental challenges [174]. Epidemiological studies report a steady increase in ASD prevalence worldwide. Its etiology is multifactorial, involving genetic susceptibility, environmental exposures, and immune dysregulation [175,176,177,178,179,180].

### 9.1. BBB Alterations in ASD

Evidence suggesting BBB involvement in ASD is increasing, although it remains largely indirect. Transcriptomic and postmortem studies have reported alterations in genes related to vascular development and endothelial markers [181,182], while genetic analyses have identified ASD-associated variants linked to adhesion and junctional proteins [183]. However, direct functional assessments of BBB permeability in ASD are still scarce.

Some immunological studies have interpreted the presence of autoantibodies against brain endothelial cells and increased levels of astrocytic markers such as GFAP and S100β as potential indicators of BBB dysfunction [184,185,186,187]. Nevertheless, these markers lack specificity and do not provide definitive evidence of barrier disruption. More direct indications of altered BBB integrity come from animal models, particularly valproic acid-induced ASD models, which show increased BBB permeability [188,189,190]. However, extrapolation of these findings to human ASD must be approached cautiously due to model-specific effects and pharmacological confounders.

### 9.2. Endothelial NO Signaling and Vascular Regulation

Immune dysregulation and chronic low-grade inflammation are frequently reported in ASD and may influence vascular and endothelial function [191,192]. Clinical studies have documented elevated circulating levels of pro-inflammatory cytokines, including IL-1β, IL-6, IL-8, IL-12p40, and TNF-α, in children with ASD, sometimes correlating with symptom severity and behavioral alterations [56,193,194,195,196,197,198,199]. These cytokines are known to activate intracellular signaling pathways that can induce NOS activity and increase NO production in endothelial and other neurovascular cells.

Furthermore, several environmental risk factors have been clinically identified, with the majority occurring during gestation. Maternal autoimmunity confers a significantly increased risk for neurodevelopmental disorders in offspring, including ASD [200]. Notably, inflammation during gestation has been identified as a significant risk factor for ASD and other neurological disorders [201,202]. In support of these findings, animal models of maternal immune activation (MIA) with various immune initiators have revealed ASD-relevant behaviors and have provided evidence of innate immune activation in MIA offspring, suggesting that early exposure to maternal inflammation may be inappropriately priming the fetal immune response and may lead to future dysfunction of this arm of the immune system [203,204,205]. Although these findings suggest that inflammatory signaling could influence neurovascular development, the precise mechanisms linking immune activation, endothelial function, and BBB regulation in ASD remain poorly understood.

### 9.3. Astrocytic NO Signaling and BBB Regulation

Astrocytic activation is frequently reported in ASD and may influence BBB function. Postmortem analyses of ASD brain tissue reveal increased astrocyte activation in several regions, including the cortex, white matter, and cerebellum [195]. Because astrocytes are central regulators of BBB stability through their perivascular endfeet, inflammatory activation of astrocytes may alter astrocyte–endothelial interactions and neurovascular homeostasis. Astrocytes also express multiple nitric oxide synthase isoforms, and inflammatory signaling can increase astrocytic NO production. However, whether astrocyte-derived NO contributes to BBB dysfunction in ASD has not yet been directly investigated.

Because astrocytes are central regulators of BBB stability through their perivascular endfeet, inflammatory activation of astrocytes may alter astrocyte–endothelial interactions and neurovascular homeostasis. Astrocytes also express multiple nitric oxide synthase isoforms, and inflammatory signaling can increase astrocytic NO production. However, whether astrocyte-derived NO contributes to BBB dysfunction in ASD has not yet been directly investigated.

### 9.4. Microglial NO Signaling and Neurovascular Inflammation

Microglial activation is another hallmark reported in ASD and may represent an important source of inflammatory mediators and nitric oxide within the brain [206]. Activated microglia release cytokines, reactive oxygen species, and nitric oxide, which can influence neuronal function and potentially affect BBB stability. Although neuroinflammation has been widely documented in ASD, the specific contribution of microglial NO signaling to neurovascular alterations remains unclear.

### 9.5. Integrative Perspective

NO signaling plays an important role in brain development and synaptic regulation, and dysregulated NO production has been implicated in ASD-related neuronal dysfunction. Strong mechanistic evidence comes from studies of SHANK3-associated ASD, where loss-of-function mutations lead to increased calcium influx, activation of neuronal nitric oxide synthase (nNOS), and excessive NO production [207,208,209,210]. These changes promote nitrosative stress and widespread alterations in the neuronal S-nitrosoproteome, affecting proteins involved in synaptic transmission and glutamatergic signaling [38,211].

Although substantial evidence links NO signaling and S-nitrosylation to neuronal dysfunction in ASD, their role in cells of the blood–brain barrier remains largely unexplored. Based on current knowledge, it is biologically plausible that S-nitrosylation could also modify proteins in endothelial cells, astrocytes, or microglia, thereby influencing neurovascular regulation. However, direct evidence connecting S-nitrosylation to BBB dysfunction in ASD is currently lacking and represents an important area for future investigation.

## 10. Integrative Perspective Across Neuropsychiatric and Neurodevelopmental Disorders

**Figure 2:**
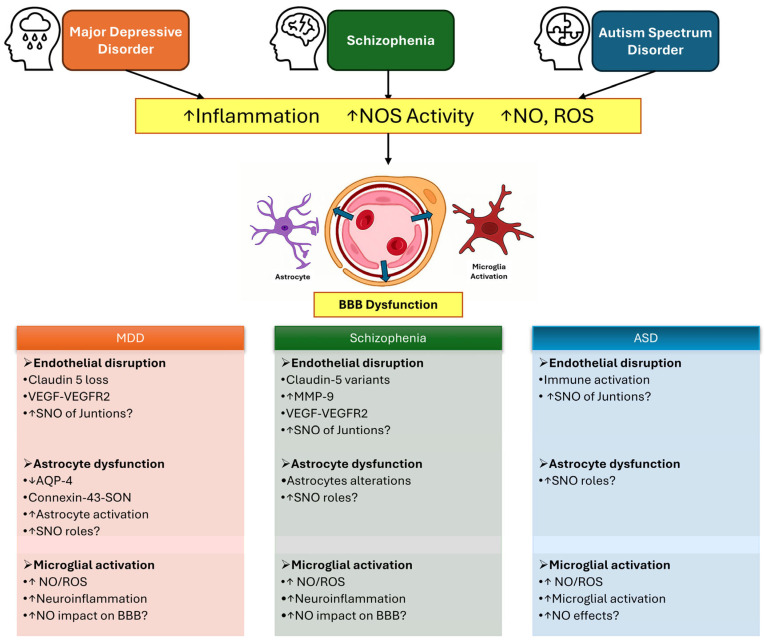
NO–S-nitrosylation in neurovascular unit dysfunction across MDD, schizophrenia, and ASD. Chronic stress and inflammation increase pro-inflammatory cytokines, activating nitric oxide synthases (NOS) in endothelial cells, astrocytes, and microglia, leading to elevated nitric oxide (NO) and S-nitrosylation (SNO). These pathways may alter endothelial junctions, astrocyte endfoot function, and microglial activation, contributing to blood–brain barrier (BBB) dysfunction. Although the mechanisms differ across disorders, a common framework links inflammation-driven NO signaling to neurovascular alterations, while the specific role of S-nitrosylation at the BBB remains to be fully established.

Across MDD, schizophrenia, and ASD, converging evidence suggests that BBB dysfunction may represent a shared—albeit heterogeneous—feature of disease pathophysiology (Figure 2). While the extent and nature of BBB alterations differ among these conditions, a common theme emerges in the interplay between inflammation, neurovascular unit NVU cell dysfunction, and redox signaling.

Pro-inflammatory cytokines, frequently elevated in all three disorders, can activate intracellular signaling cascades in endothelial cells, astrocytes, and microglia, leading to the induction of NOS and increased NO production. In this context, NO acts as a pleiotropic mediator capable of modulating vascular tone, cellular communication, and inflammatory responses. However, under conditions of sustained stress or immune activation, excessive NO production may contribute to oxidative and nitrosative stress, thereby affecting BBB integrity.

At the cellular level, endothelial dysfunction, subtle alterations in tight junction composition, and changes in vascular signaling pathways are observed across MDD and schizophrenia and are increasingly suggested in ASD. Astrocytic alterations—including impaired endfeet coverage and dysregulation of proteins such as aquaporin-4—may further compromise BBB stability and neurovascular coupling. In parallel, microglial activation contributes to a pro-inflammatory milieu, amplifying NO and reactive oxygen species production and potentially reinforcing barrier dysfunction. Together, these processes support a model in which BBB alterations arise from the coordinated dysregulation of multiple NVU components rather than from isolated endothelial failure.

Evidence supporting a role for S-nitrosylation is strongest in neuronal systems, particularly in ASD, where alterations in the S-nitrosoproteome have been well characterized—but remains limited in BBB-forming cells. Nonetheless, given the sensitivity of junctional proteins, cytoskeletal elements, and signaling molecules to redox regulation, it is reasonable to hypothesize that S-nitrosylation could influence key processes governing BBB permeability and stability.

Despite these converging observations, important differences must be acknowledged. BBB alterations in MDD and schizophrenia appear to be subtle, region-specific, and potentially reversible, whereas in ASD they may reflect early-life or developmental perturbations linked to immune activation and neurodevelopmental trajectories. Moreover, the heterogeneity within each disorder suggests that BBB dysfunction and NO-related mechanisms may be relevant only to specific patient subgroups.

**Figure 3:**
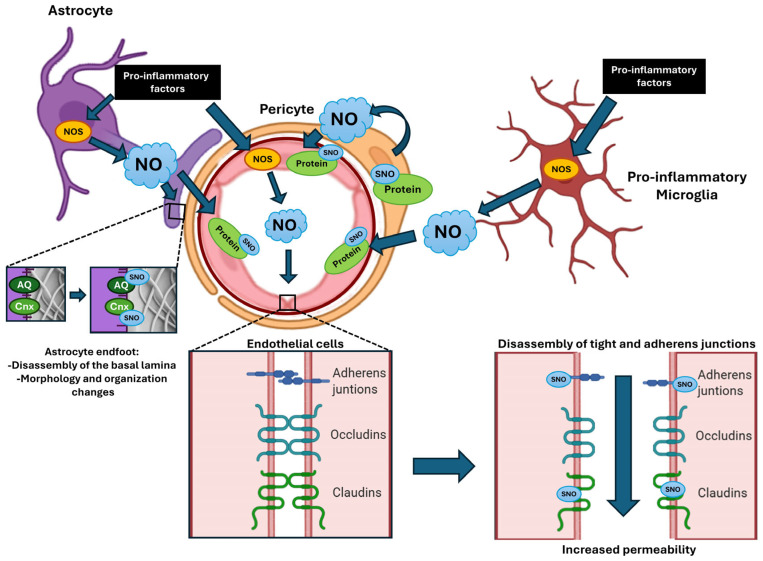
Overview of the NO production mechanism and S-nitrosylation of protein. NO can be generated by different isoforms of NOS in astrocytes, microglia, pericytes, and endothelial cells. The activation of NOS stimulated by proinflammatory factors enhances NO production. Increased levels of NO promote the S-nitrosylation of proteins, which in endothelial cells, results in the disassembly of tight and adherens junctions, leading to increase vascular permeability. In astrocytes, S-nitrosylated proteins such as aquaporins (AQ) and connexins (Cnx) can promote disassembly of the endfoot to the basal lamina and morphological changes.

Overall, current evidence supports a unifying framework in which inflammation-driven NO signaling and redox imbalance contribute to neurovascular dysregulation across distinct brain disorders. However, direct mechanistic links between NO-dependent S-nitrosylation and BBB dysfunction remain largely unproven. Figure 3 shows possible roles of S-nitrosylation in different cell types in the NVU. Addressing this gap will require integrative approaches combining neuroimaging, in vivo models, and redox proteomics to identify disease-relevant S-nitrosylated targets within the NVU. Such efforts may ultimately clarify whether NO signaling represents a shared therapeutic target across neuropsychiatric and neurodevelopmental conditions.

## 11. Current Limitation and Future Direction

This work compiles evidence regarding NO and protein S-nitrosylation role in the BBB within neuropsychiatric conditions. Research on this specific topic is relatively recent, resulting in a limited amount of evidence. Most mechanisms are derived from cell and animal experiments, and clinical sample sizes are small. Additionally, the technology for detecting S-nitrosylation has not yet been standardized, which affects academic objectivity.

Nonetheless, there is enough consensus to justify further investigation into how protein S-nitrosylation at the BBB contributes to neuropsychiatric conditions. Based on current knowledge, it is biologically plausible that S-nitrosylation could also modify proteins in endothelial cells, astrocytes, pericytes and microglia, thereby influencing neurovascular regulation. Future researchers should aim to clarify NO-dependent mechanisms at the BBB and to further understand cellular behavior under conditions of stress and elevated NO production. Enhancing our knowledge of these NO-dependent molecular and cellular signaling pathways may reveal new therapeutic targets, potentially alleviating the neuroinflammatory burden associated with neuropsychiatric conditions.

## Data Availability

No new data were created or analyzed in this study. Data sharing is not applicable to this article.

## Abbreviations

AQP-4	Aquaporin-4
ASD	Autism spectrum disorder
ASL	Arterial sin labeling
BBB	Blood–brain barrier
CNS	Central nervous system
COVID-19	Coronavirus-19
DAMPs	Damage associated molecular patterns
DCE-MRI	Dynamic contrast- enhancer magnetic resonance imaging
eNOS	Endothelial nitric oxide synthase
GFAP	Glial fibrillary acidic protein
GSNO	S-nitrosoglutathione reductase
HMGB1	High-mobility group box 1
HPA	Hypothalamic–pituitary–adrenal
IL-12	Interleukin 12
IL-12p40	Interleukin 12 p40 subunit
IL-1α	Interleukin 1 alpha
IL-1β	Interleukin 1 beta
IL-6	Interleukin 6
IL-8	Interleukin 8
INF-γ	Interferon gamma
iNOS	Inducible nitric oxide synthase
KO	Knock out
LPS	Lipopolysaccharide
MDD	Major depressive disorder
MMP-9	Matrix metalloproteinase 9
MPPs	Matrix metalloproteinase
MRI	Magnetic resonance imaging
nNOS	Neuronal nitric oxide synthase
NO	Nitric oxide
NOS	Nitric oxide synthase
NUV	Neurovascular unit
PAF	Platelet active factor
rCBF	Regional cerebral blood flow
ROS	Reactive oxygen species
sGC-PKG	Soluble guanyl cyclase-protein kinase G
SHANK3	SH3 and multiple ankyrin repeat domains protein 3
SNO	S-nitrosylation
SNRI	Serotonin-norepinephrine reuptake inhibitors
SSRI	Selective serotonin reuptake inhibitors
TGF-β	Transforming growth factor beta
TNF-α	Tumor necrosis factor alpha
VASP	Vasodilator stimulated phosphoprotein
VEGF	Vascular endothelial growth factor
VEGFR-2	Vascular endothelial growth factor receptor 2
ZO-1	Zonula occludens 1
ZO-2	Zonula occludens 2
